# Aliskiren/amlodipine as a single-pill combination in hypertensive patients: subgroup analysis of elderly patients, with metabolic risk factors or high body mass index

**DOI:** 10.3109/21556660.2012.762367

**Published:** 2012-12-25

**Authors:** Christoph Axthelm, Christian Sieder, Franziska Meister, David Pittrow, Edelgard Kaiser

**Affiliations:** 1 Cardiologicum, PirnaGermany; 2 Clinical and Regulatory Affairs, Novartis Pharma GmbH, NuernbergGermany; 3 Institut für Klinische Pharmakologie, Technische Universität DresdenGermany; 4 Clinical and Regulatory Affairs, Novartis Pharma GmbH, NuernbergGermany

**Keywords:** Amlodipine, Aliskiren, Arterial hypertension, Combination therapy, Diabetes mellitus, Elderly, Metabolic risk factors, Olmesartan

## Abstract

**Aims:**

Blood pressure (BP) reduction in hypertensive patients is more difficult to achieve in the elderly or in the presence of comorbidities. We aimed to investigate the efficacy of the single-pill combination (SPC) aliskiren/amlodipine in hypertensive elderly patients, patients with high body mass index (BMI), with at least one metabolic risk factor, and/or type 2 diabetes mellitus (DM).

**Methods:**

In an open-label non-randomized study, patients not adequately controlled by previous treatment with the SPC olmesarten 40/amlodipine 10 (phase 1) were switched to the SPC aliskiren 300/amlodipine 10 (phase 2). The present post-hoc analysis investigated BP reduction in phase 2 in the named subgroups. The EudraCT identifier was 2009-016693-33, ClinicalTrials.gov identifier NCT01113047.

**Results:**

Of the 187 patients not adequately controlled in phase 1 and thus treated with the SPC aliskiren 300/amlodipine 10 in phase 2, 69 were of advanced age (≥65 years), 74 or 89 were overweight or obese (BMI 25.0–29.9 kg/m^2^ or ≥30 kg/m^2^, respectively), 91 had metabolic risk factors (without DM) and 41 had DM. At the beginning of phase 2, depending on the subgroup, baseline SBP was 168–169 mmHg and DBP 103–104 mmHg. After 4 weeks of treatment with aliskiren 300/amlodipine 10, SBP/DBP was lowered by −5.1/−4.8 mmHg in the total cohort, by −5.5/−5.1 mmHg in elderly patients, by −6.7/−5.5 in overweight and by −4.2/−4.5 mmHg in obese patients, by −6.4/−4.7 mmHg in patients with metabolic risk factors without DM, and by −3.3/−5.0 mmHg in DM patients. Limitations include low sample size, limited treatment duration and the fact that the post-hoc defined groups were not mutually exclusive.

**Conclusions:**

In this study reflecting clinical practice, the aliskiren/amlodipine combination achieved effective BP reduction in elderly patients or with metabolic comorbidities, including DM that might be more difficult to treat. This consistent BP lowering pattern facilitates everyday care of patients who receive aliskiren/amlodipine.

## Introduction

Hypertension is an established major risk factor for an array of cardiovascular complications such as myocardial infarction or stroke, and is associated with a substantial increase in risk of premature death^1^. The great majority of hypertensive patients require drug treatment to control their condition, usually in the form of combinations with drugs of different mode of action and synergistic efficacy^2–5^. Current guidelines advise the use of combination therapy not only in second-line, but also in first-line therapy^5–7^. In addition, the ESH/ESC Guidelines recommend that in patients at higher risk, goal BP should be achieved promptly, which supports initial combination therapy and quicker adjustment of doses. Common clinical variables that influence prognosis should be used to stratify patient risk, including among others, higher age, abdominal obesity, diabetes mellitus (DM) or other metabolic risk factors.

The dihydropyridine calcium channel blocker (CCB) amlodipine, administered at doses of 5–10 mg once daily, is a direct peripheral arterial vasodilatator that reduces peripheral vascular resistance, and may reduce left ventricular hypertrophy^8–10^. The basis for approval of the single-pill combination (SPC) olmesartan/amlodipine (Sevikar), was formed by a factorial dose-finding study in patients with mild-to-moderate hypertension^11^, a long-term safety study^12^ and two randomized, double-blind studies in patients with moderate-to-severe hypertension who were not adequately treated on one component alone^13,14^.

The direct renin inhibitor aliskiren inhibits the renin–angiotensin–aldosterone system at its rate-limiting step. Aliskiren was tested in patients with essential hypertension in a large development program as monotherapy or in double and triple combination with other classes of anti-hypertensive drugs^15,16^. A number of studies with aliskiren investigated the drug in special patient groups (elderly, obese, components of the metabolic syndrome, see Table 1).

**Table 1.** Overview of studies with aliskiren in obese, elderly, or metabolic impaired patients.

Author and references	Design	Subgroup *n*	Duration (weeks)	Aliskiren schedule (mg/day)	Control drug schedule (mg/day)	ΔSBP/DBP vs. baseline in the aliskiren group (mmHg)	ΔSBP/DBP vs. baseline in the control group (mmHg)
**Obese (BMI ≥30)**							
Schmieder^37^	r, pc, db, mc	396	12	150 → 300	HCT 12.5 → 25	16.7/12.3	12.2/9.1
			52	+AMLO 5 → 10	+AMLO 5 → 10	19.9/15.5	17.6/13.3
Jordan^36^	r, pc, db, mc	489	8	HCT 25 + 150 → 300	HCT 25 + irbesartan 150 → 300	15.8/11.9	15.4/11.3
			8		HCT 25 + AMLO 5 → 10		13.6/10.3
			8		HCT 25		8.6/7.9
Whaley-Conell^38^	r, c, db, mc	386	8	150 → 300 + HCT 12.5 → 25	Ramipril 5 → 10	28.1/10.1	16.6/3.6
Braun-Dullaeus^39^	r, c, db, mc	482	8	150 → 300 + AMLO 5 → 10	AMLO 5 → 10	35.9/15.1	30.0/10.9
Weir^61^	Pooled meta-analysis	905	8–12	300	Placebo	13.9/11.1	4.9/6.1
**Elderly (aged 65+ years)**							
Verdecchia^54^	r, c, db, mc	355	8	75	Lisinopril 10	8.4/4.5	10.2/6.3
				150		7.1/3.6	
				300		8.7/3.9	
Duprez^55^	r, c, db, mc	901	12	150 → 300	Ramipril 5 → 10	14.0/5.1	11.6/3.6
			22	+HCT 12.5 → 25	+HCT 12.5 → 25	19.6/8.2	17.3/7.3
			36	+HCT 12.5 → 25 + AMLO 5 → 10	+HCT 12.5 → 25 + AMLO 5 → 10	20.0/8.2	18.1/7.0
Yarows^61^	r, pc, db, mc	1797	8	150 → 300	Placebo	14.5/8.6	5.1/5.3
				+VAL 160 → 320	VAL 160 → 320	19.0/12.6	12.2/11.0
Gradman^62^	Pooled	704	8–12	150	Placebo	13.4/11.7	4.4/8.1
				300		14.9/12.3	
Littlejohn^63^	o, mc	101	54	150 → 300 + AMLO 5 → 10 (+HCT 12.5 → 25)		30.4/17.6	
Basile^64^	r, c, db, mc	54	8	150 → 300 + HCT 12.5 → 25		34.2/24.0	
Schmieder^56^	o, prospective	7399	52	150 → 300 ± other antihypertensive drug	ACEi/ARBs No ACEI/ARBs	19.6/7.6	15.5/6.3 16.3/6.6
					No ACEi/ARB		9.6/6.7
Villa^53^	r, pc, db	754	8	75	Placebo	13/5	8/4
				150		15/6	
				300		14/7	
**Diabetes mellitus**							
Uresin^47^	r, c, mc	560	8	150 → 300	Ramipril 5 → 10	14.7/11.3	12.0/10.7
Taylor^49^	Meta-analysis	1417	8–12	150	Placebo	13.2/10.4	6.9/7.8
				300	14.8/12.2		
Townsend^48^	r, c, db, mc	860	8	150 → 300 (+HCT 12.5 → 25)	AMLO 5 → 10	28.8/9.9	26.2/9.0
Verpooten^51^	o, prospective	759	26	150 → 300	25.1/11.3		
Sowers^65^	Meta-analysis	1035	8–12	150	Placebo	12.7/7.8	6.8/5.7
				300		15.2/11.6	
Zeymer^50^	o, prospective	4242	52	150 → 300	ACEi/ARB	18.2/7.7	14.7/6.5
					Non ACEi/ARB		14.2/6.3
Braun-Dulleaus^66^	r, db, c, mc	219	8	150 → 300 + Am 5 → 10	AMLO 5 → 10	34.4/15.2	30.5/12.3
**Metabolic syndrome**							
Weinberger^43^	r, c, db, mc	197	8	150 → 300 + AMLO 5 → 10	AMLO 5 → 10	36.4/15.0	28.5/10.5
Krone^41^	r, c, db	141	12	300	Irbesartan 300	13.8/7.1	5.8/2.8
White^67^	Meta-analysis	2903	8–12	150	Placebo	13.3/10.4	5.1/5.8
				300		14.8/11.3	
Lacourciere^68^	r, db, mc, c	613	8	ALIS 300 + AMLO 10 + HCT 25	ALIS 300 + AMLO 10	36.2/20.0	28.9/16.9
					ALIS 300 + HCT 25		26.6/13.9
					AMLO 10 + HCT 25		29.7/16.5
Ferdinand^44^	r, db, mc, c	284	8	ALIS 300 + AMLO 10	ALIS 300 + AMLO 10 + HCT 25	28.2/11.9	34.9/14.9
Gradman^42^	Pooled	166 238	8 -- 12	150 300	Placebo	15.0/11.4 15.1/11.7	5.0/5.9

Δ, difference; c, (active) controlled; db, double blind; HCT, hydrochlorothiazide; mc, multicenter; r, randomized; o, open; pc, placebo controlled; ACEI, angiotensin converting enzyme inhibitor; ALIS, aliskiren; AMLO, amlodipine; ARB, angiotensin receptor blocker; HCT, hydrochlorothiazide; VAL, valsartan. → indicates titration step (e.g. 150 → 300: from 150 mg/day to 300 mg/day).

Aliskiren and amlodipine have similarly long half-lives (aliskiren 40 h and amlodipine 30–50 h), which allows constant BP reduction throughout the entire dosing interval^17^ and beyond^18–20^. Besides complimentary mechanisms of action, aliskiren is capable to compensatorily down-regulate the rise in plasma renin activity following the addition of amlodipine^21^. The fixed combination of the two drugs (Rasilamlo) was investigated in a factorial study^22^, a long-term study^23^ and in add-on studies in patients not adequately responding to the monotherapy^24,25^, which led to the approval of the SPC aliskiren/amlodipine^26^.

In the present post-hoc analysis of a previously published prospective non-randomized study^27^ we aimed to investigate the BP lowering effect of the SPC aliskiren/amlodipine in special patient groups with hypertension that typically present in everyday practice and may pose a particular challenge to treating physicians owing to comorbidity, comedication or complexity of the disease: patients of advanced age, high body mass index (BMI) (overweight, obesity), with metabolic risk factors and/or with DM.

## Patients and methods

### Study design

The AWESOME study (Aliskiren in Combination With Amlodipine in Hypertensive Patients Not Responding to Angiotensin Receptor Blocker Plus Amlodipine) was performed between May and October 2010 as a multicenter, open-label, non-randomized single-arm study in 38 centers in Germany. The study was approved by the ethics committee of the State Chamber of Physicians in Saxony in Dresden and the responsible health authority (BfArM, Federal Institute for Drugs and Medical Devices). All patients provided written informed consent prior to inclusion in the study. The study was conducted according to the ethical principles of the Declaration of Helsinki. It was registered in the ClinicalTrials.gov database under NCT01113047 and in EudraCT under 2009-016693-33.

**Figure 1.**
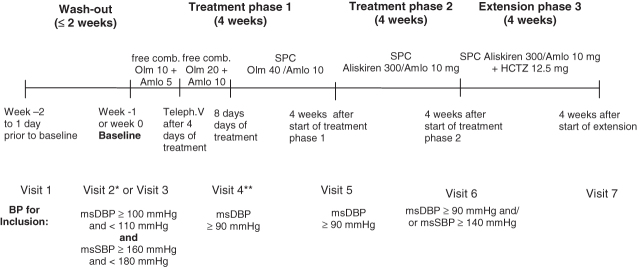
Study design. *Visit 2 was performed only in patients with previous antihypertensive therapy. **Patients with an office mean sitting DBP<90 mmHg at an unscheduled visit after visit 4 or at visit 5 were discontinued from the study. Dotted box highlights study phase from which data were analyzed for this paper.

The study consisted of three phases, as shown in Figure 1: after wash out, patients with mean sitting diastolic blood pressure (msDBP) 100–109 mmHg and mean sitting systolic blood pressure (msSBP) 160–179 mmHg at visit 3 (baseline) were included into a 4-week treatment phase 1 with forced titration to the SPC olmesartan 40 mg/amlodipine 10 mg; in non-responders (msDBP at trough ≥90 mmHg) this was followed by a subsequent 4-week treatment phase 2 with the SPC aliskiren 300 mg/amlodipine 10 mg once daily. This period was followed in non-responders (msDBP ≥90 mmHg and/or msSBP ≥140 mmHg at trough) by an optional 4-week extension with the SPC aliskiren 300 mg/amlodipine 10 mg/HCT 12.5 mg once daily. The aliskiren/amlodipine and aliskiren/amlodipine/HCT combinations were investigational formulations manufactured by Novartis for this study, whereas the olmesartan/amlodipine film tablets used were marketed products. The first 60 patients who were eligible and agreed to participate were included in the extension (results reported previously)^27^. Study drugs were to be taken orally in the morning. Compliance was assessed by pill count.

### Patients

Male and female patients aged ≥18 years with uncomplicated moderate essential hypertension as defined above were eligible for participation. Patients were excluded from the core study, if they presented with DBP ≥110 mmHg or SBP ≥180 mmHg at any time between visit 1 and baseline, or if they could not discontinue all antihypertensive medication safely for a period of up to 2 weeks.

Further important exclusion criteria comprised: controlled BP levels on current antihypertensive medication (DBP <90 mmHg and SBP <140 mmHg) at visit 1; known hypertensive retinopathy; heart failure in NYHA II–IV; secondary hypertension; refractory angina pectoris; significant hepatic or renal disease; DM [type 1 or poorly controlled type 2, defined as a fasting blood glucose >200 mg/dL (>11.1 mmol/L)]; second- or third-degree heart block without a pacemaker; significant arrhythmia or valvular heart disease; history of transient ischemic attack, stroke, hypertensive encephalopathy, myocardial infarction; contraindications for the study medication, and use of concomitant medications known to significantly affect the metabolism of the study drug or BP, pregnant or breast feeding women. Main exclusion criteria for the extension study were premature discontinuation during the core study, hypersensitivity or contraindications to diuretics, in particular to HCT.

### Efficacy and safety parameters

The primary efficacy parameter of this trial was the change in trough msDBP between visit 5 (start of treatment phase 2) and visit 6 (end of treatment phase 2), and in addition further BP parameters were assessed (including changes in SBP, responder rates, etc.) as well as safety and tolerability. BP was recorded using a calibrated standard sphygmomanometer and appropriate size cuff, in accordance with the 2005 AHA Committee Report on blood pressure measurement^28^. BP values were measured three times at each visit, and the mean of all three sitting measurements recorded.

Patient aged ≥65 years were defined as elderly. The diagnosis DM in these hypertensive patients was according to physician’s individual assessment. Overweight was defined as BMI 25.0–29.9 kg/m^2^, obesity as BMI ≥30 kg/m^2^. The condition ‘at least one metabolic risk factor’ was present, if serum glucose was ≥5.56 mmol/l, LDL cholesterol ≥4.16 mmol/l, or triglycerides were ≥2.28 mmol/l.

### Safety and tolerability

The safety information included frequency of adverse events (AE), results of physical examinations, data on body weight, and laboratory evaluations. Vital signs were assessed as part of the efficacy evaluations. All laboratory samples, obtained in a fasting state, were sent to a central laboratory for analysis.

### Statistical analysis

The safety population of the respective phase comprised the sample of all patients who took at least one dose of the study medication, the intention-to-treat population (ITT) of all patients from the safety population in phase 2 who had at least one evaluation of the primary efficacy parameter after visit 5 (baseline phase 2).

The present post-hoc analysis was performed in subgroups of ITT patients with certain diagnoses as provided by demographic criteria (elderly patients), physician diagnoses (DM) in the context of clinical and laboratory criteria (metabolic risk factors including obesity). Patients suitable for several subgroups were analyzed in all these groups. The main analysis was performed descriptively on the mean change in trough SBP/DBP between visit 5 and visit 6 (treatment phase 2). The interpretation of all secondary parameters was explorative.

## Results

### Patient flow

A total of 439 patients were screened of which 97 were not eligible (e.g., BP criterion not fulfilled) and 342 patients were enrolled in phase 1. A total of 187 patients were switched to aliskiren 300 mg/amlodipine 10 mg at the beginning of phase 2. In that phase, 74 patients were overweight, 89 were obese, 69 were aged 65 years and above (elderly), and 132 had at least one metabolic risk factor (91 without DM and 41 with DM).

Baseline characteristics in the total cohort and in the subgroups are shown in Table 2. With the exception of two patients, all were Caucasians, mean age overall was 60.5 years, and 58.3% of patients were male. Mean age was higher in the group of patients with DM (64 years), while the gender distribution did not vary much across subgroups.

**Table 2.** Baseline characteristics in all 3 phases, including subgroups in phase 2.

Parameter	Phase 1	Phase 2								Phase 3
	Safety population^1^	All subgroups								Safety population
		ITT population	BMI 25–29.9	BMI ≥30	Met risk	Met risk no DM	Met risk DM yes	Age <65 years	Age ≥65 years
	OLME 40/ AMLO 10	ALIS 300/ AMLO 10								ALIS 300/AMLO 10/HCT 12.5
Number of patients	342	187	74	89	132	91	41	118	69	65
Age (years) mean (±SD)	60.4 (10.9)	60.5 (10.7)	60.9 (10.9)	59.4 (10.9)	60.7 (10.3)	59.2 (10.6)	64.0 (8.6)	54.3 (8.1)	71.1 (4.6)	60.8 (10.8)
Gender – *n* (%)										
Male	182 (53.2)	109 (58.3)	46 (62.2)	54 (60.7)	79 (59.8)	55 (60.4)	24 (58.5)	69 (58.5)	40 (58.0)	46 (70.8)
Female	160 (46.8)	78 (41.7)	28 (37.8)	35 (39.3)	53 (40.2)	36 (39.6)	17 (41.5)	49 (41.5)	29 (42.0)	19 (29.2)
Race – *n* (%)										
Caucasian	340 (99.4)	185 (98.9)	73 (98.6)	88 (98.9)	132 (100)	91 (100)	41 (100)	116 (98.3)	69 (100.0)	64 (98.5)
Black/other	2 (0.6)	2 (1.1)	1 (1.4)	1 (1.1)	0 (0.0)	0 (0.0)	0 (0.0)	2 (1.7)	0 (0.0)	1 (1.5)
Weight (kg) mean (±SD)	88.5 (19.3)	90.6 (20.1)	82.5 (10.4)	103.4 (19.7)	90.4 (19.7)	87.8 (16.4)	96.3 (25.0)	94.4 (22.6)	84.0 (12.7)	91.1 (19.0)
SBP (mmHg) mean (±SD)	166.6 (6.2)	167.9 (5.3)	167.9 (5.2)	167.1 (5.3)	168.3 (5.4)	168.0 (5.1)	168.9 (5.8)	166.8 (4.9)	169.7 (5.5)	168.3 (5.2)
DBP (mmHg) mean (±SD)	103.1 (3.0)	103.6 (2.6)	103.5 (2.7)	103.4 (2.5)	103.7 (2.6)	103.8 (2.7)	103.6 (2.5)	103.6 (2.4)	103.6 (2.9)	103.3 (2.3)

BMI, body mass index; DBP, diastolic blood pressure; DM, diabetes mellitus; ITT, intent-to-treat; HCT, hydrochlorothiazide. Met risk, at least one metabolic risk factor present; SBP, systolic blood pressure. Values are means (SD, standard deviation), or percentages (*n* (%))

### Blood pressure

**Figure 2.**
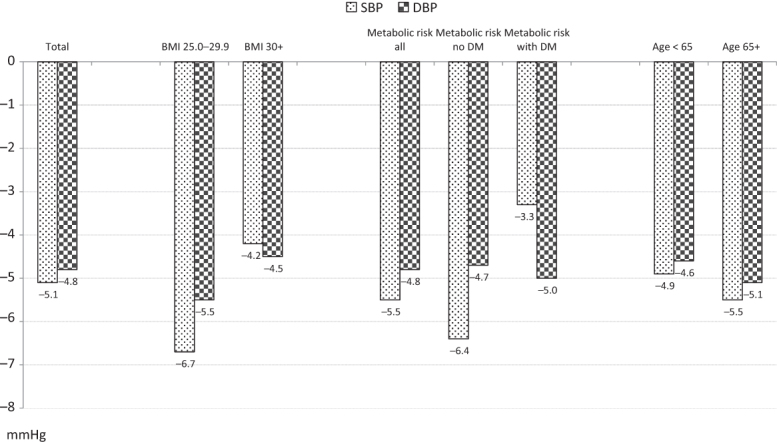
Blood pressure reduction on aliskiren/amlodipine in the total cohort (ITT population) and in subgroups in phase 2 (week 8 compared with week 4).

SBP and DBP at the beginning of the treatment phases are displayed in Table 2, bottom. Mean baseline BP at the beginning of phase 2 in the total patient cohort was 168/104 mmHg, and it was nearly identical in the various subgroups. SBP and DBP reduction at end of phase 2 was −5.1/−4.8 mmHg in the total cohort, −5.5/−5.1 in the elderly, −6.7/−5.5 in overweight patients, −4.2/−4.5 in obese patients, and −5.5/−4.8 mmHg in metabolic risk patients (−6.4/−4.7 without DM, −3.3/−5.0 with DM; Figure 2).

### Tolerability and safety

Overall, 19 patients (10.1%) in phase 2 experienced at least one AE. No deaths or serious adverse events occurred. The most frequently observed AEs were ‘peripheral edema’ in seven (3.7%) patients, ‘edema’ in three (1.6%) patients and ‘eczema’ in one (0.5%) patient. All other AEs occurred only in one or two patients at maximum. Changes in laboratory values generally were infrequent, in accordance with the known profiles of the drugs. Details have been reported elsewhere^27^. AE were not analyzed by subgroups in view of the low patient numbers.

## Discussion

The present subgroup analysis shows that treatment with the aliskiren/amlodipine SPC achieves meaningful BP reductions in elderly or obese patients, in metabolic risk patients or DM patients. These BP reductions were in line with the BP reduction in the overall study population.

Such patients are frequent in everyday care, and they may present a particular challenge to treating physicians. In a cross-sectional point prevalence study of 45,000 unselected consecutive patients in a representative nationwide sample of primary care physicians in Germany (HYDRA), 28% had a BMI ≥30.0 kg/m^2^ ^29^. Overall prevalence of hypertension, defined as BP ≥140/90 mmHg or on antihypertensive medication, was 60.6%, in grade 1 obesity 72.9%, in grade 2 obesity 77.1%, and in grade 3 obesity 74.1%. The odds ratio for good BP control (<140/90 mmHg) in diagnosed and treated patients was 0.8 in overweight patients compared with normal weight, 0.6 in grade 1, 0.5 in grade 2, and 0.7 in grade 3 obese patients^29^. While patients with overweight/obesity are not specifically considered in the current ESC hypertension guidelines, those with metabolic syndrome are. Although no implication is made that it is a pathogenetic entity, metabolic syndrome is specifically mentioned as it constitutes a cluster of risk factors often associated with high BP which markedly increases cardiovascular risk^30–35^. At least five definitions with slight or moderate deviations from each other have been proposed by international organizations or by expert groups^30–35^. In our study, a more general approach was chosen, as patients with at least one ‘metabolic risk factor’ were analyzed in the respective group.

With respect to age, in elderly patients with uncomplicated hypertension, treatment should be initiated gradually, while in those with higher risk, goal BP should be achieved more promptly, which favors initial combination therapy and quicker adjustment of doses^5^.

As high age, high BMI and metabolic problems including DM are highly prevalent, the aliskiren monotherapy and aliskiren/amlodipine combination studies have included substantial fractions of such patients and found conclusive and consistent effects on BP in the various subgroups (Table 1).

In obese patients, aliskiren-based treatment was highly effective and well-tolerated in patients who failed first line-treatment with a thiazide^36^, and (with optional addition of amlodipine) had a substantially lower incidence of hypokalemia (1 vs. 14%) compared with thiazide-based therapy^37^. In the same study, aliskiren provided a stronger antihypertensive effect compared with HCT^37^. In the ATTAIN study, the aliskiren/HCT combination was more effective than ramipril^38^, and a subanalysis of an aliskiren/amlodipine combination study showed similar BP lowering in obese or non-obese patients^39^. Recently, Boschmann highlighted aliskiren's prolonged BP-lowering effect following discontinuation, and showed that the drug penetrates adipose and skeletal muscle tissue at levels that are apparently sufficient to reduce tissue RAS activity^40^.

With regard to patients with metabolic syndrome, the Krone *et al*. study suggested that aliskiren 300 mg may offer advantages to irbesartan 300 mg^41^. In the pooled analysis by Gradman *et al*. no differences in the BP lowering effects between women with or without metabolic syndrome were noted^42^. Weinberger *et al*. reported that the aliskiren/amlodipine combination was more effective than amlodipine monotherapy in African-Americans with obesity or metabolic syndrome^43^. In a subgroup analysis of the ASCENT study, the dual aliskiren/amlodipine combination was as effective as the triple aliskiren/amlodipine/HCT combination in lowering BP among high-risk US minority patients with cardiometabolic syndrome^44^.

In patients with DM, studies on aliskiren (alone or in combination with angiotensin receptor blockers) mainly focused on the assessment of antiproteinuric/nephroprotective effects rather than the antihypertensive effects^45,46^. However, the studies by Uresin *et al*. (aliskiren vs. ramipril)^47^ and Townsend *et al*. (aliskiren/HCT vs. amlodipine)^48^ showed beneficial BP lowering effects of aliskiren in diabetic patients. Also, a pooled analysis of aliskiren depicted the same effect in diabetic versus non-diabetic patients^49^. Interestingly, prospective registry data from the 3A Registry and the DRIVER study supported this finding in unselected patients^50,51^.

In elderly patients, aliskiren exposure is modestly increased^52^. A significant dose relationship for the BP lowering effect was found in the elderly, with no evidence of dose-related increases in adverse events^53,54^. Compared with a ramipril-based regime, aliskiren lowered BP significantly stronger in elderly patients with systolic hypertension^55^. A substantial BP reduction over the long term has further been substantiated in the 3A Registry with 15,000 unselected patients^56^.

Overall, the present analyses of the various subgroups in the AWESOME study, taken together with evidence from the aliskiren/amlodipine study program and other randomized studies indicate that the use of the combination leads to comparable efficacy in various subgroups. This could facilitate clinical management of the diverse hypertensive population. The overall results of the study show that the switch of patients not adequately controlled by the SPC olmesartan 40 mg/amlodipine 10 mg once daily to aliskiren 300/amlodipine 10 mg benefits about one-third of patients (36.4%) that can be treated to target at the end of phase 2 (week 8). For non-normalizers in phase 2, the addition of a thiazide diuretic in a SPC further led to a significant reduction of SBP and DBP.

Some methodological limitations should be considered when interpreting the results of the present analysis. Patients could be part of more than one subgroup (populations were not mutually exclusive). An open-label study, in particular without a control arm, can be compromised by unknown bias of various types^57^. The multiple subgroup analyses were specified post-hoc and carried out descriptively, and performed to assess the consistency of a treatment effect among various patient characteristics. Thus, the typical problems of subgroup analyses (in particular multiplicity) do not apply here^58^. However, the sample size of the current study was low and the treatment duration limited. Larger and longer-term studies are needed for definitive conclusions. Also for the assessment of safety, much larger datasets are needed^59^.

## Conclusion

The SPC aliskiren 300 mg/amlodipine 10 mg had consistent BP lowering effects in various subgroups of patients with stage 2 hypertension. The BP lowering effect was in line with the achieved BP lowering effect in the total population of this study, and with various reports of other randomized controlled trials on this combination.

## Transparency

### Declaration of funding

The study was funded by Novartis Pharma GmbH.

### Declaration of financial/other relationships

C.S. and F.M. are employees of Novartis Pharma GmbH Germany. E.K. is a former employee of Novartis. D.P. has received consultancy honoraria from Novartis.

## Acknowledgments

We acknowledge the cooperation and commitment of all investigators and their staff, who made the present trial possible.

These results were presented, among others, at the 21st Annual European Meeting of Hypertension 17–20 June 2011, Milan, Italy.
